# Genomics as a time capsule: insights from *Oreobates chiquitanus* type specimens

**DOI:** 10.1186/s12864-026-12984-5

**Published:** 2026-06-06

**Authors:** Yannis Schöneberg, Marcel Nebenführ, Martin Jansen, Axel Janke, Sven Winter

**Affiliations:** 1 Institute for Ecology, Evolution, and Diversity, Goethe University, Frankfurt am Main, Germany; 2 Senckenberg Biodiversity and Climate Research Centre (BiK-F), Frankfurt am Main, Germany; 3 Department of Biogeography, Trier University, Trier, Germany; 4 Department of Terrestrial Zoology, Senckenberg Research Institute and Nature Museum, Frankfurt am Main, Germany; 5 Noel Kempff Mercado Natural History Museum, Santa Cruz de la Sierra, Bolivia; 6 LOEWE-Centre for Translational Biodiversity Genomics (TBG), Frankfurt am Main, Germany; 7 Faculty of Science and Technology, University of the Faroe Islands, Tórshavn, Faroe Islands

**Keywords:** Anura, Museomics, Conservation, Genome, Neotropics, Population, Inbreeding, Holotype Sequencing, Deforestation, Global Change Biology

## Abstract

**Background:**

Biodiversity is at increasing risk, and amphibians are the most threatened vertebrate class, with 40.7% of species globally at risk. For amphibians, the primary cause for their decline is habitat loss. The scale of this global issue becomes most evident in so-called deforestation hotspots, such as the Chiquitano Dry Forest in Bolivia, which harbours a highly diverse fauna and flora with many species still awaiting formal description. Only recently, the frog *Oreobates chiquitanus* has been described from a single location within this forest. Since then, it was discovered at only two additional sites. An ongoing logging initiative at the type locality led to the deforestation of the sampling site, even before the species was formally described. Ongoing logging activities in the vicinity of all known sites raise questions about the species’ fate. Here, we provide a comprehensive genetic definition of the species and try to assess the genetic diversity and conservation value of the type population.

**Results:**

We sequenced all available type specimens of *Oreobates chiquitanus*, providing a reference genome from one of the paratopotypes and mitochondrial assemblies for all type specimens. We further show that all known populations of this species are near recent logging initiatives. The reconstruction of the demographic history indicates that the population was recovering from a dip roughly 300kya. The estimates of relatedness and heterozygosity imply a genetically vital population.

**Conclusions:**

This study exemplifies the importance of collaboration of natural history collections and genomic initiatives like TBG (Translational Biodiversity Genomics) for a better understanding of the anthropogenic impacts in times of global change, to identify and describe biodiversity, to raise awareness, and inform potential conservation measures. We not only provide a comprehensive and permanent molecular definition for *O. chiquitanus* but also assess the genetic diversity of the population. Therefore, this study provides an important baseline for future studies of genetic erosion in this species. Furthermore, it illustrates the risk of biodiversity loss in the Chiquitano Dry Forest and signifies the need for conservation efforts.

**Supplementary Information:**

The online version contains supplementary material available at 10.1186/s12864-026-12984-5.

## Background

Today, the loss of biodiversity is an ubiquitous and global phenomenon. Biodiversity declined by 2% to 10% during the last century [1], and an estimated one million species worldwide face extinction [2]. The five main drivers of shifts in biodiversity are closely linked to human activities (starting with those that have the most impact): Land and sea use change, direct exploitation of organisms, climate change, pollution, and invasive species [2]. Agriculture expansion, the most widespread form of land use change, affects a third of the world´s land surface and therefore has an immense impact on terrestrial ecosystems and species [2, 3]. Habitat loss through legal and illegal agriculture expansion is also the single most important driver of amphibian decline, the most threatened vertebrate class, with 40.7% of species globally threatened [4]. The regional extent of today’s biodiversity loss from agricultural expansion might become tangible by examining tropical deforestation hotspots, where rapid, widespread habitat destruction occurs, affecting large but unknown numbers of individual plants and animals across a wide range of taxa in highly diverse biomes.

**Fig. 1:**
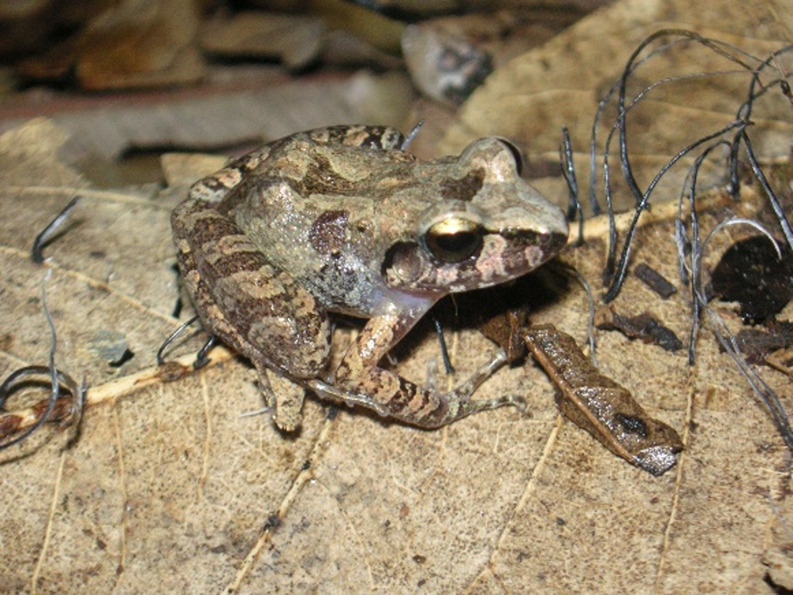
Photo of *Oreobates chiquitanus* holotype (SMF88497) from San Sebastián, Provincia Nuflo de Chávez, Department Santa Cruz, Bolivia

**Fig. 2:**
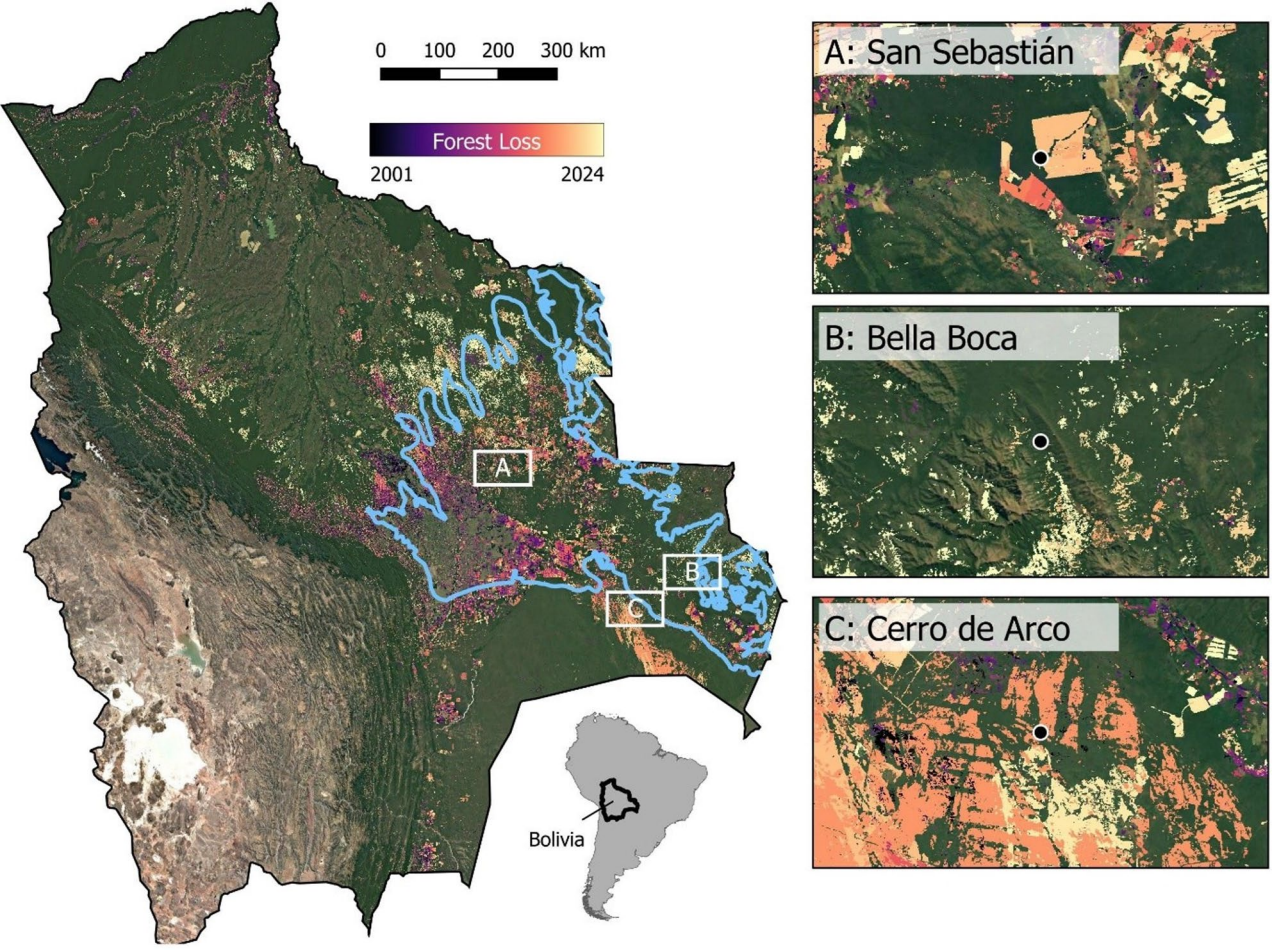
Logging activity in the Chiquitano Dry Forest in relation to known locations for *O. chiquitanus*. Map of the three known occurrence locations of *O. chiquitanus* in the Chiquitano Dry Forest in regard to forest loss in the Chiquitano region (blue outline) between 2001 and 2024. The small maps show the forest loss at the occurrence sites of *O. chiquitanus* in more detail. The maps demonstrate that the Chiquitano Dry Forest is heavily affected by ongoing logging campaigns, and all sites from which *O. chiquitanus* is known are in close proximity (< 10 km) to recent logging activities (orange to light yellow areas; see text for details, satellite image source: Google Satellite; forest loss: Global Forest Watch, version 1.12 [20]

Bolivia, for instance, has ranked among the top 3 countries globally in terms of primary forest loss since 2020 [5]. In October 2024, 10 to 11 million ha (9% to 10% of the country) were damaged by wildfires that threatened critical ecosystems, including the Amazon rainforest, Chaco dry forests, the Pantanal wetland, and the Chiquitano Dry Forest (CDF) [6–9]. Only recently, the frog species *Oreobates chiquitanus* (Fig. 1, Pansonato, Motta, Cacciali, Haddad, Strüssmann & Jansen [10]) was described from a single locality in this forest. Since then, this species was only discovered at two other localities (Fig. 2), and, according to Pansonato et al. (2020) [10] is assumed to be associated with and restricted to the Chiquitano Dry Forest. An ongoing logging initiative of the local landowner at the type locality led to the deforestation of the sampling site, even before the species description could be formally published.

On one hand, this reflects the overall situation of the entire Chiquitano Dry Forest, which is one of the deforestation hotspots worldwide. This forest is endemic in the eastern lowlands of Bolivia and harbours a highly diverse fauna and flora, but remains poorly studied, with many species still waiting for formal description. The fate of many species is questionable because it is linked to that of the Chiquitano Dry Forest ([6, 11]; Kümmerle et al., submitted). Since 1985, nearly one quarter of the area was reduced due to agriculture, with rates of deforestation and wildfires accelerating since 2020 ([11, 12]; Kümmerle et al., submitted).

On the other hand, the destruction of the type locality of *Oreobates chiquitanus* makes obtaining further material from the type population impossible, impeding future taxonomic or molecular work. Type specimens are the physical reference that define a species, allowing comparison. Tying a name to specific specimens ensures taxonomic stability and consistency in species identification and classification. Therefore, type specimens and the knowledge of the type locality are of particular importance, offering a valuable source of data for (bio)geographical and spatial studies, or as the source of more reference material for further, subsequent, or reproduced studies [13]. Unfortunately, while types are deposited and archived in a natural history collection and are therefore preserved for comparative or reproductive studies, type localities are subject to change due to external influences, and their representativeness over time is not guaranteed.

Institutions are trying to combat these challenges and make their collections more accessible and public by digitising voucher specimens [14, 15], e.g. by creating 3D scans [16], or holotype sequencing [17–19] as permanent digital resources. With genetic barcoding and more recently, high-throughput sequencing, the molecular definition of a species becomes more and more important, as in particular genomes, pose a comprehensive reference to biodiversity, its history and adaptation. Although there is no agreed-upon definition yet, holotype sequencing aims to conserve as much of the holotype’s genetic information as possible. This can economically be done by whole genome shotgun sequencing employing common short read sequencing technologies at a low to medium sequencing depth [17–19]. This data can then be assembled de-novo (i.e. without a reference) to generate an at least fragmented draft assembly. While the resulting draft assembly is usually of low contiguity, it provides a permanent and degradation-free resource for future taxonomic, evolutionary and comparative work, either as it is, or mapped onto a higher-quality assembly of the same species.

In the face of the recent landscape changes at the type locality and in the Chiquitano Dry Forest in general, as well as the significance of the specimens, we herein aim to preserve the genetic definition and diversity of the recently discovered frog *Oreobates chiquitanus* [10]. We take holotype sequencing a step further by generating a contig level reference genome assembly from one of the paratopotypes of *O. chiquitanus* using long-read sequencing and re-sequenced all type specimens using short read data, including the holotype. The sequenced genomes are a permanent genetic definition for this species, irrespective of the fate of the type population and the species in general. Additionally, the generated data enables a first assessment of the genomic diversity at the type population prior to destruction, providing an important basement to study genomic erosion in the future and to inform conservation action.

## Methods

### Sampling and sequencing

For this study, muscle tissue samples from the four type specimens (SMF88497 – SMF88500) of *Oreobates chiquitanus* at the Senckenberg Museum in Frankfurt am Main, Germany, were collected. The specimens were collected in the ‘‘Centro de Investigaciones Ecologicas Chiquitos’’, San Sebastián, Provincia Nuflo de Chávez, Departamento Santa Cruz, Bolivia (Fig. 2) in November 2010 and were previously used to describe the species [10]. The specimens were stored in 70% ethanol at room temperature. We took the tissue samples from the upper leg, a body region of low taxonomic interest, to preserve the taxonomic significance of the specimens. For the assembly of the reference genome, we extracted high-molecular-weight DNA from the type specimen SMF 88498 using the Qiagen DNeasy Blood & Tissue kit. We then prepared a PacBio SMRTbell Express Template Prep Kit 2.0 and sent it for CLR sequencing on a PacBio Sequel II (Pacific Biosciences, Menlo Park, CA, USA). Additionally, 150 bp paired-end libraries with an average insert size of 350 bp were prepared for all four samples using the NEBNext Ultra II DNA Library Prep Kit for Illumina (New England Biolabs Inc., Ipswich, MA, USA) and sequenced on an Illumina NovaSeq6000 at Novogene (Cambridge, UK) to an approximate 30-fold coverage for the specimen of the genome assembly and 10X for the re-sequenced specimens.

### Assembly

We assembled the reference genome using Flye v2.9 [21] on standard settings, including one iteration of long-read polishing. To correct for errors in the CLR reads, we performed short-read polishing in three steps. First, the short-read data were trimmed and filtered using TrimGalore v0.6.7 [22], which itself uses cutadapt v3.4 [23]. Second, we mapped the short-read data against the assembly using bwa-mem2 index and mem v2.2.1 [24] and samtools sort v1.14 [25], removed PCR duplicates using picard MarkDuplicates v2.26.6 [26], and created an index of the assembly using samtools v1.14 [25]. Last, we ran DeepVariant v1.2 [27] to identify single nucleotide polymorphisms (SNPs) and used this information to create a polished consensus sequence using the bcftools v1.14’s view, tabix, and consensus commands [25]. The quality of the final assembly was assessed using BUSCO v5.2.2 [28] on the tetrapoda_odb10 dataset [29], Quast v5.0.2 [30], and blobtoolkit v4.1.5 [31].

To annotate repetitive sequences in the genome, we created a custom *de novo* repeat library using RepeatModeler v2.0.1 [32] and combined it with the vertebrates repeat database from RepBase [33]. Afterwards, we annotated the repeats using RepeatMasker v4.1.2 [34]. The repeat landscape was created using RepeatMasker’s calcDivergenceFromAlign.pl script, and the results were plotted using ggplot2 v3.5.1 [35] in R v4.4.1, omitting RNA repeats [36].

Additionally, we assembled the mitochondrial genomes for each specimen from the short-read data using GetOrganelle v1.7.5.3 [37]. The resulting assemblies were aligned using MAFFT v7.525 [38] and we calculated the raw genetic distances using the dna.distance command of the package Ape v5.8.1 [39] in R v4.4.1 [36]. We circularized and annotated each mitochondrial genome using MitoZ annotate v3.6 [40]. The annotations were checked for completeness.

### Population analyses

We assessed the genome-wide heterozygosity, historical population sizes, inbreeding coefficient and relatedness to get a glance at the population’s genetic diversity. For all analyses except the heterozygosity calculations, we used the trimmed and filtered short-read data mapped to the genome of *O. chiquitanus*. The reads were mapped to the assembly using bwa-mem2 index and mem v2.2.1 [24] and samtools sort v1.14 [25]. PCR duplicates were removed using picard MarkDuplicates v2.26.6 [26], and indices of the read alignment files were created using samtools v1.14 [25]. In order to calculate the inbreeding coefficients, we called genotype likelihoods from the mapped bam files using ANGSD v0.935 [41] (angsd -gl 2 -domajorminor 1 -snp_pval 1e-4 -domaf 1 -minmaf 0.05 -doGlf 3) and subsequently ran ngsRelate v2 [42].

We further estimated the historical effective population size (Ne) of the type population of *O. chiquitanus* using a Sequentially Markovian Coalescent (PSMC) analysis. At first, we had to generate reference-based assemblies by calling the consensus sequence from using the BCFtools v1.14 command consensus [25]. We filtered the resulting files to remove sites with mapping quality < 30 and read depth < 10 or > 2× the mean depth. Finally, we ran PSMC v0.6.5-r67 [43] with 25 iterations, 100 rounds of bootstrapping. We assumed a generation time (g) of 3 years, similar to *Lynchius simmonsi* (formerly *Oreobates simmonsi*) [44] and a mutation rate (m) of 0.3 × 10^− 8^ substitutions per nucleotide and generation [45].

For estimating the heterozygosity, we applied a slightly different approach. Unfortunately, no genome-wide estimates for heterozygosity in anurans were available for comparison. To calculate heterozygosity estimates for additional frog species, we first downloaded all available frog genomes from NCBI that had at least 10x short-read sequencing depth. This means we calculated heterozygosity for the 4 *O. chiquitanus* specimens and 5 individuals total from 3 additional species (Supplement Table S1). To avoid coverage bias during SNP calling, we randomly reduced short-read coverage to 10X using seqtk sample v1.3 (https://github.com/lh3/seqtk) for all samples. Then, we mapped the short-read data against the respective species’ reference genome using bwa-mem2 index and mem v2.2.1 [24] and Samtools fixmate, sort, and removed PCR duplicates using picard MarkDuplicates v2.26.6 [26] and created an index of the assembly using samtools index v1.14 [25]. To check the mapping quality, we ran qualimap bamqc v2.2.2a [46]. Then, GATK v4.4.0.0 [47] was used to call and filter SNPs, using its commands CreateSequenceDictionary, HaplotypeCaller, and GenotypeGVCFs with setting the parameter --all_sites true in order to get all callable sites. The resulting data was then filtered using bcftools filter v1.21 [25] with the following filtering settings: -e ‘QUAL < 20 && MQ < 20 && FORMAT/DP > 20 && FORMAT/DP < 6 && TYPE="indel"’. To estimate the heterozygosity, we first used bcftools stat on the variant calling data to count the number of SNPs and the overall callable sites. Then we divided the number of SNPs by the overall number of sites to get the genome-wide heterozygosity.

To visualise the threat posed by logging to this species, we created a map showing the occurrence of *O. chiquitanus* in relation to logging activity. The occurrence points are the type locality, from which the specimens examined in this study originated, and two additional known localities [10, 48]. We used satellite image data from a Google Satellite, and combined it with forest loss data from Global Forest Watch version 1.12 [20].

## Results

Genome assembly.

The final assembly is approximately 3.4 Gbp in length and has a contig N50 of approximately 332 kbp. The BUSCO analysis recovered 84.3% Complete BUSCOs, 6.4% fragmented, and 9.3% missing (Table 1). This indicates a rather contiguous assembly, considering the poor taxonomic coverage of anura in the tetrapoda_odb10 dataset [29]. The Blobtools analysis shows that most contigs cluster together and are assigned to Chordata. Only a few contigs are assigned to other taxa. However, due to the lack of amphibian genomes available at the time of analysis, these annotations are likely false positive hits. Overall, the blobtools analysis indicates an assembly with negligible contamination (Fig. S1-2).

**Fig. 3:**
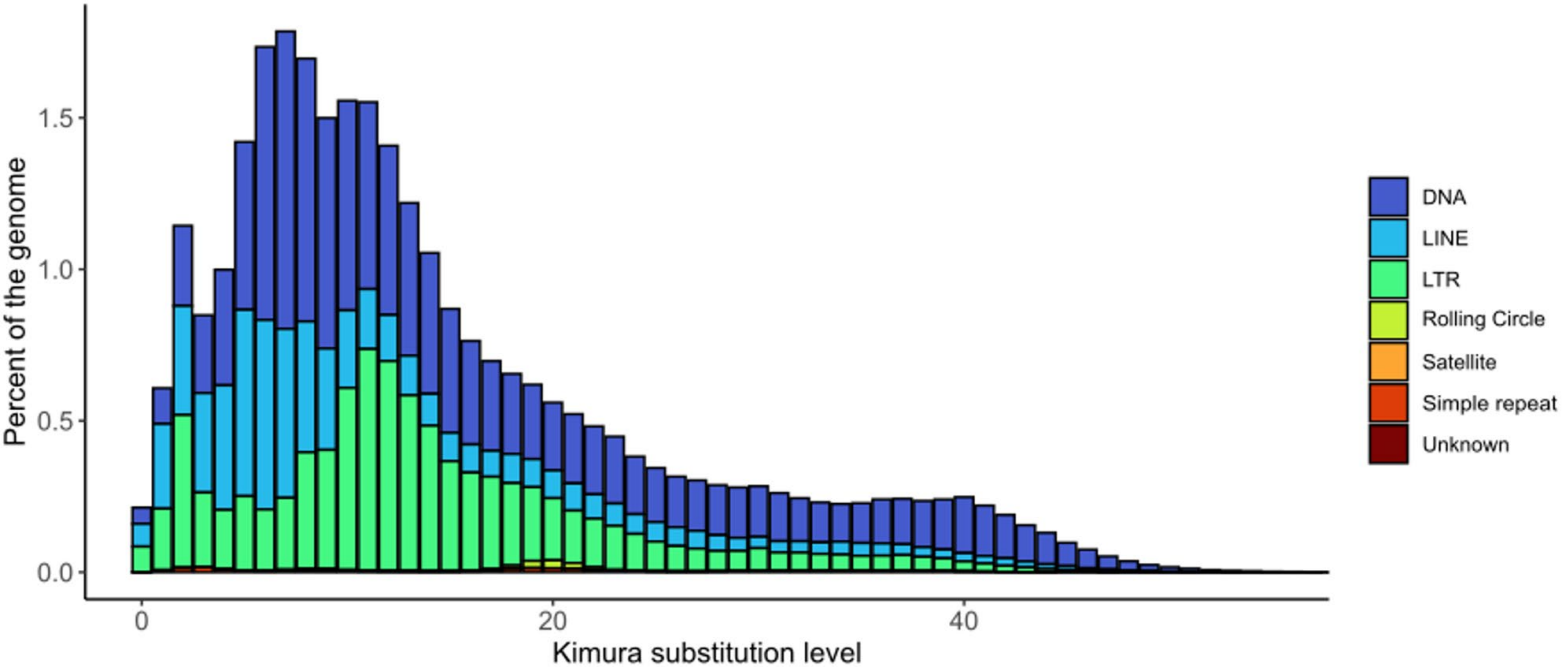
Repeat landscape for *Oreobates chiquitanus*. It shows that DNA repeats were active even in the more distant past (high Kimura substitution levels) whereas LTRs and LINES were more recently active

The genome of *O. chiquitanus* consists of 72.92% of repetitive sequences, to a large proportion, these are interspersed repeats (71.06% of the assembly), and only very few are Small RNA, simple or low complexity repeats, and microsatellites (1.86% of the assembly, Table S2). The low number of tandem repeats is probably influenced by our contig-level assembly, and repeat content in general is probably higher. The repetitive sequences identified as interspersed repeats are mostly unknown repeats that could not be assigned to any repeat family. The repeat landscape shows DNA repeats even with high Kimura substitution levels, likely indicating that they were constantly active (Fig. 3). In contrast, LTRs were most active in the near past, and most LINEs have low Kimura substitution levels, indicating recent activity (Fig. 3).

For subsequent analyses, we mapped the short-read data of each *O. chiquitanus* specimen against the reference assembly. In every specimen, > 99% of the reads mapped against the reference, with a mean mapping quality larger than 38 (Table S3). This indicates excellent mapping qualities, well-suited for downstream analyses.

**Table 1:** Statistics for the final reference assembly. The statistics were calculated using Quast and BUSCO (see Methods for details)

Assembly	final assembly
# contigs	34,963
Largest contig	2,659,982
Total length	3,446,581,503
GC (%)	45.31
N50	332,777
N75	149,332
L50	2873
L75	6690
# N’s per 100 kbp	17.98
BUSCO:	
Complete [%]	84.3
Single copy [%]	82.7
Multi copy [%]	1.5
Fragmented [%]	6.4
Missing [%]	9.3
Markers	5310

### Population analyses

**Fig. 4:**
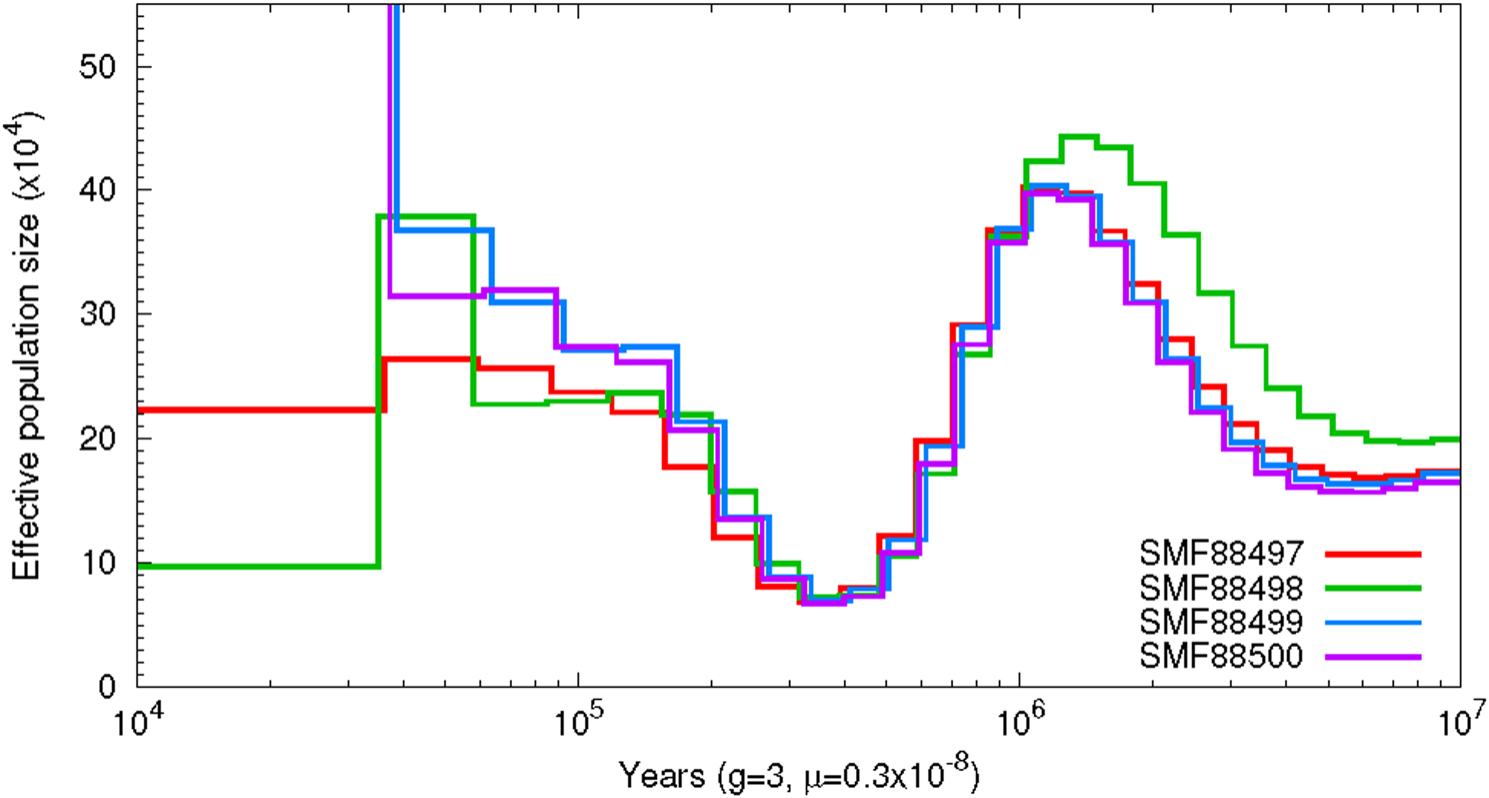
PSMC-Analysis for the four *O. chiquitanus* specimens. All specimens show similar curves, indicating that they share the same population level history. The analysis estimates an ancient population expansion followed by a decline in more recent times. The peak of the expansion is roughly 1 Mya and the lowest effective population size was reached around 300 kya. After this decline there was a recovery of effective population size

We reconstructed the historical population size of the type population using a PSMC analysis. This analysis shows there was a peak in population size approximately 1 Mya, followed by a decline until 300 kya, with the population size recovering in more recent times (Fig. 4). The population decline roughly coincides with increasing climatic fluctuations during the Pleistocene [49]. However, whether there exists a causal relationship remains speculative.

The Jacquard coefficients J9 were estimated to be 1 in all specimen combinations, whereas the remaining coefficients J1 – J8 were 0. This indicates that all specimens were unrelated to each other. The inbreeding coefficient based on these Jacquard coefficients (rab; Fa; Fb; IR_1_2; IR_2_1; Frat.; FDiff) also indicates no relation between the examined specimens. However, the R0, R1, and KING analyses consistently had values that identified the specimens as third-degree relatives (Tale 2).

**Table 2:** Selected inbreeding coefficients calculated using ngsRelate2. The Jacquard-Indices and derived statistics clearly indicate that the tested individuals were unrelated. However, R0, R1, and KING values identified all individuals to be third-degree relatives. Abbreviations: IR – inbreeding relatedness. Specimen codes in columns a and b are as follows: 0 - SMF88500; 1 - SMF88497; 2 - SMF88498; 3 - SMF88499

a	b	J9	J1–8	rab	Fa	Fb	IR_1_2	IR_2_1	Frat.	FDiff	R0	R1	KING
0	1	1	0.000	0.000	0.000	0.000	0.000	0.000	0.000	0.000	0.312	0.285	0.072
0	2	1	0.000	0.000	0.000	0.000	0.000	0.000	0.000	0.000	0.253	0.293	0.096
0	3	1	0.000	0.000	0.000	0.000	0.000	0.000	0.000	0.000	0.245	0.296	0.099
1	2	1	0.000	0.000	0.000	0.000	0.000	0.000	0.000	0.000	0.314	0.260	0.067
1	3	1	0.000	0.000	0.000	0.000	0.000	0.000	0.000	0.000	0.320	0.258	0.065
2	3	1	0.000	0.000	0.000	0.000	0.000	0.000	0.000	0.000	0.241	0.280	0.097

We generated genome-wide heterozygosity estimates for *O. chiquitanus* and three additional frog species for comparison (Table 3). Of these species, the genome-wide heterozygosity was highest in the single specimen of *S. bombifrons* with 1.119%. *O. chiquitanus* followed with 0.492% heterozygosity. The specimens of *H. boettgeri* had a mean heterozygosity of 0.157%, and it was lowest in the single individual of *L. fallax* with only 0.041% of heterozygous sites.

**Table 3:** Genome wide heterozygosity results for 4 different anuran species

Species	Mean(Het)	Std	*N*(samples)
*H. boettgeri*	0.186	0.024	3
*L. fallax*	0.051	-	1
*O. chiquitanus*	0.492	0.096	4
*S. bombifrons*	1.119	-	1

**Fig. 5:**
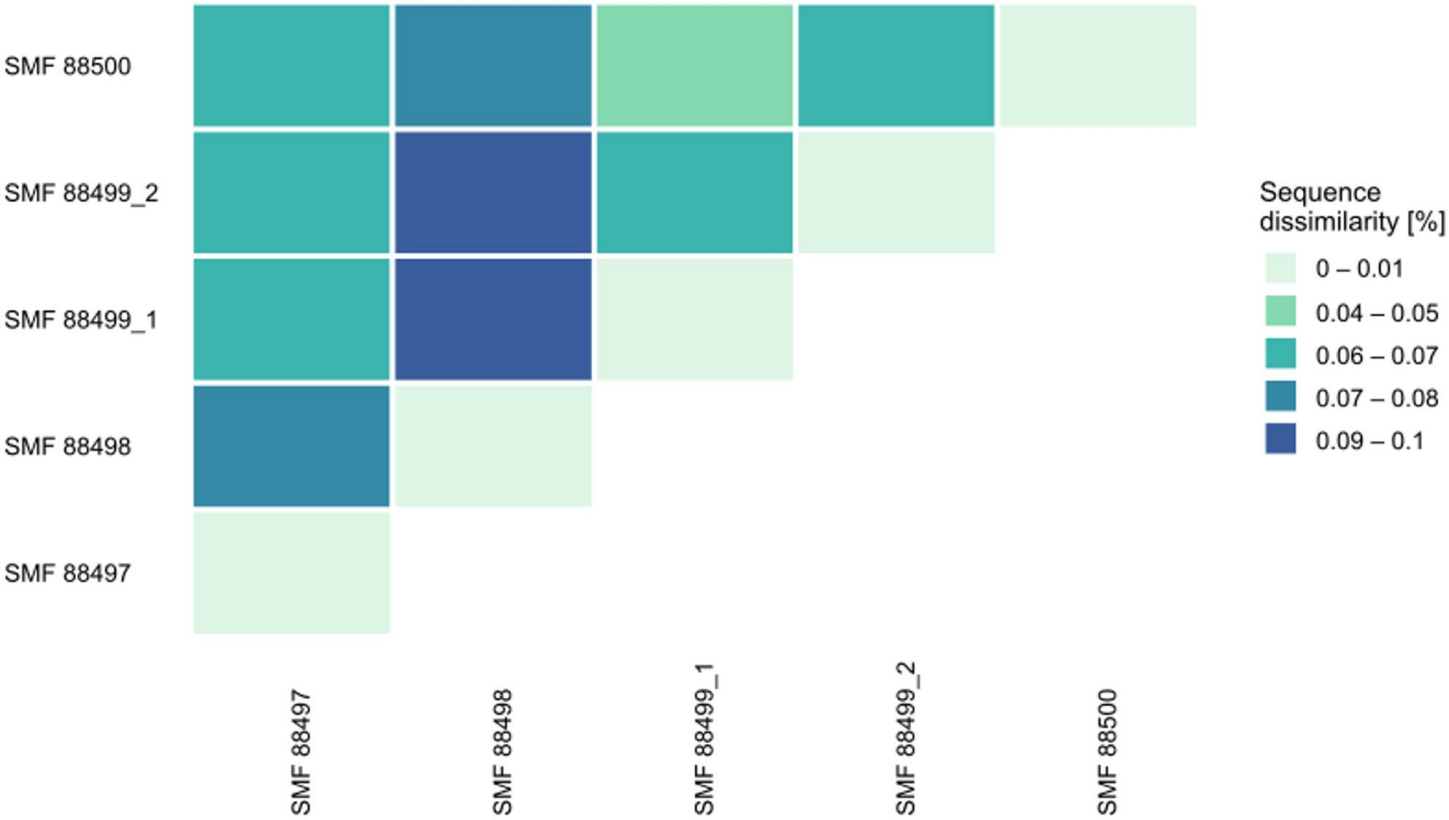
Raw sequence distances among mitochondrial genomes. For individual SMF 88499 two different assemblies were reconstructed which differed mainly by indels in the control region. As mitochondria differ between all specimens, all specimens had different mothers

We generated mitochondrial genome assemblies for each *O. chiquitanus* specimen. The resulting assemblies were all circular and ranged in length from 16,465 to 17,849 bp. For specimen SMF 88499, there were two alternative assemblies reconstructed, both of 17,850 bp length. Both sequences differed mostly by insertions or deletions in the control region (Fig. 5 & S2). In general, all specimens had differing mitochondrial genomes with the raw distances varying from 0.04% to 0.09% (Fig. 5). For each mitogenome, we annotated 13 protein-coding genes, 2 rRNAs, and 23 tRNAs, with tRNA-Leu, tRNA-Ser, and tRNA-His being duplicated.

The analysis of logging history in the Chiquitano dry forest shows that all three populations known for *O. chiquitanus* are located in close proximity (< 10 km) to large and ongoing logging initiatives (Fig. 2).

## Discussion

We sequenced all type specimens of *O. chiquitanus* and used the data to provide a reference genome. The assembly statistics indicate a comprehensive and contiguous de novo reference assembly (Table 1), and the mapping rate and quality of the resequencing data were high (Table S3). Despite being derived from museum material, with many potential sources of contamination (e.g., reuse of ethanol), the blobtools analysis indicates only negligible contamination (Fig S1-S2). The mitogenomes assembled for each specimen had 13 protein-coding genes, 2 rRNAs, and 23 tRNAs. Usually, vertebrates only possess 22 tRNAs in their mitochondria [50], and many amphibians do so [51, 52]. However, for the anuran genus *Nanorana*, mitochondrial genomes with 23 tRNAs were reported with tRNA-Leu, tRNA-Ser, and tRNA-Met being duplicated [53], whereas in our assemblies, tRNA-Leu, tRNA-Ser, and tRNA-His were duplicated. Furthermore, in other frogs, considerable structural rearrangements and gene duplications were shown [54]. Therefore, it seems structural variation in the mitochondrial genomes of amphibians is common at least in some clades. For one specimen, we assembled two different mitochondrial genomes. The assemblies differed mainly by indels in the control region and a few base substitutions in the remaining sequence. A possible explanation could be an artefact from non-functional copies of mitochondrial DNA in the nuclear genome, so-called Nuclear Mitochondrial DNA segments (NUMTs). However, the chosen assembly pipeline filters for NUMTs using a read coverage-based approach [37]. Furthermore, indels accumulated exclusively in the hypervariable control region. If the segments were NUMTs, one would expect the mutations to occur throughout the whole sequence. In other vertebrate mitochondria, indels also occurred in the control region [55–57]. Therefore, it seems likely that the alternative assembly is caused by heteroplasmy, the presence of multiple mitochondrial lineages within a single individual. This phenomenon is described in a few amphibian species [58, 59]. However, a detailed analysis of long-read and transcriptomic data would be needed to confirm heteroplasmy.

As we provide comprehensive genomic data for all type specimens of this species, this dataset represents an invaluable resource for future taxonomic and evolutionary work. Type specimens and the knowledge of the type locality are of particular importance for taxonomic research and evolutionary studies: They are the physical reference and definition for a species. By sequencing all type specimens and providing a reference genome from one of the paratopotypes, we provide a highly comprehensive and permanent genetic characterization of the species, which became especially important with the destruction of the type locality.

Although we cannot assess the current state of the type population, local logging campaigns fragmented the landscape and likely also the population. The locality, formerly covered with primary forest, was destroyed in 2020 by a logging campaign of the landowner, ongoing until today (pers. comm. Martin Jansen). The genetic diversity of a given population degrades only over time, and can survive short bottlenecks in population size [60]. Yet ongoing logging initiatives in the local area are threatening the survival of this unique population. Also, both other known localities from which this frog is known are located less than 10 km from ongoing and large-scale logging campaigns. With the logging activity soaring in the Chiquitano Dry Forest (11, 12; Kümmerle et al., submitted), *O. chiquitanus* is likely confronted with drastic land use changes throughout its whole distribution area (Fig. 2), illustrating the need for conservation efforts in the Chiquitano Dry Forest to slow the loss of biodiversity.

As the fate of the frog is unclear, we wanted to provide a strong resource for future studies on genomic erosion, informing conservation measures. In the past, studies have shown that genomic data greatly facilitate conservation biology. De novo genomes themselves already show the full architecture of a species’ genetic information, but also allow for large-scale population genomics studies with the help of reference-guided assemblies, to infer population-wide genetic markers of a species, crucial for their protection [61, 62]. Therefore, we assessed the genetic diversity of the type population prior to the location’s deforestation. The PSMC analysis indicates that the population size was recovering after a dip roughly 300 kya (Fig. 4). However, the results of a PSMC analysis do not allow reliable statements on the more recent history of the population. Unfortunately, the sample size did not allow for a reliable estimation of more recent population sizes [63]. Therefore, we also calculated different measures of genetic diversity. Our analyses of relatedness showed varying results based on the employed method. The Jacquard-based indices inferred all individuals to be completely unrelated from each other, whereas the King and R1, R2 analyses indicate the individuals might be third-degree relatives (Table 2). Due to the small sample size and resulting noise in allele frequency estimates, the Jacquard coefficients might not have been able to identify non-zero Identity-By-Descent states [64], while the third-degree relationship estimated by King could also reflect local population structure. One could argue that our dataset is biased because all specimens were collected on the same night and at the same location, originating from the same or closely related clutches. However, the difference in mitochondrial haplotypes suggests unrelated mitochondrial and, therefore, different maternal lineages (Fig. 4). Also, the comparably high heterozygosity indicates a genetically diverse population (Table 3). Therefore, we think even though specimens were collected near each other, it is unlikely that the specimens were closely related or inbred, but we cannot exclude background relatedness. Hence, we conclude that the type population was likely genetically diverse, or at least not impoverished before the destruction of its location.

## Conclusions

Our results provide a permanent and comprehensive genomic resource of all type specimens and hence the molecular fingerprint of *Oreobates chiquitanus*. Our study, therefore, highlights the importance of natural history museums in times of global biodiversity loss as they harbour valuable collections of both extant and extinct species. It underscores how this type of data was used to capture information on the genetic structure and diversity of *the* type population of O. chiquitanus prior to the deterioration and loss of its habitat. The case of *O. chiquitanus* emphasizes the benefits of using genomic data in conservation biology and exemplifies the need for conservation efforts to slow down the loss of Biodiversity in the Chiquitano Dry Forest.

## Supplementary Information

Supplementary Material 1.

## Data Availability

All genetic data generated for this study were deposited at NCBI’s GenBank under the Bioproject PRJNA1226607 . The specimens are located in the Herpetological Collection of the Senckenberg Natural History Museum Frankfurt, Senckenberganlage 25, 60325 Frankfurt, Germany under collection numbers SMF 88497-88500.
