# Supplementary material for: Genomics as a time capsule: insights from Oreobates chiquitanus type specimens
Source: BMC Genomics. 2026 Jun 6;27:525. doi: 10.1186/s12864-026-12984-5 (PMC13242125; doi:10.1186/s12864-026-12984-5)
Supplement: Supplementary file 1 — Supplementary Material 1. [file 12864_2026_12984_MOESM1_ESM.docx]

# Supplement Oreobates

Table S1: NCBI-accessions for the genomes and short read data used in estimating the genome-wide heterozygosity in multiple anuran species.

| Organism Name | Assembly Accession | Illumina Accessions |
| --- | --- | --- |
| Hymenochirus boettgeri | GCA_019447015.1 | SRR10362925, SRR10362926, SRR9309200, SRR9309201 |
| Spea bombifrons | GCA_027358695.2 | SRR9140157 |
| Leptodactylus fallax | GCA_947044405.1 | SRR13806021 |

Table S2: Repetitive elements identified in the genome assembly of Orebates chiquitanus. Abbreviations: LINE, Long Interspersed Nuclear Elements; LTR, Long Terminal Repeat elements; SINE, Short Interspersed Nuclear Elements.

| Class | Count | Masked [bp] | Masked [%] |
| --- | --- | --- | --- |
| SINEs | 67 977 | 27 546 224 | 0.8 |
| LINEs | 502 782 | 214 206 749 | 6.22 |
| LTR | 364 037 | 282 161 480 | 8.19 |
| DNA transposons | 761 993 | 308 415 738 | 8.19 |
| Rolling-circles | 6 964 | 1 372 585 | 0.04 |
| Unclassified | 6 844 049 | 1 644 268 178 | 47.71 |
| Total Interspersed | 8 547 802 | 2 449 052 145 | 71.06 |
|  |  |  |  |
| Small RNA | 57 502 | 17 998 562 | 0.52 |
| Satellites | 14 024 | 5 274 948 | 0.15 |
| Simple repeats | 309 530 | 34 103 248 | 0.99 |
| Low complexity | 35 710 | 5 550 610 | 0.16 |
|  |  |  |  |
| Total | 8 964 568 | 2 513 352 098 | 72.92 |

Table S3: Mapping statistics for the reference-based assemblies. All specimens had values above 99 % and high mapping qualities, indicating excellent reference-based assemblies.

|  | SMF 88497 | SMF 88498 | SMF 88499 | SMF 88500 |
| --- | --- | --- | --- | --- |
| Number of reads | 558 927 079 | 583 046 225 | 607 208 736 | 580 023 534 |
| Mapped reads [%] | 99.19 | 99.06 | 99.32 | 99.38 |
| Mean mapping quality | 38.5 | 41.9 | 38.7 | 38.6 |
| Mean coverage | 22.9 | 24 | 25 | 23.9 |


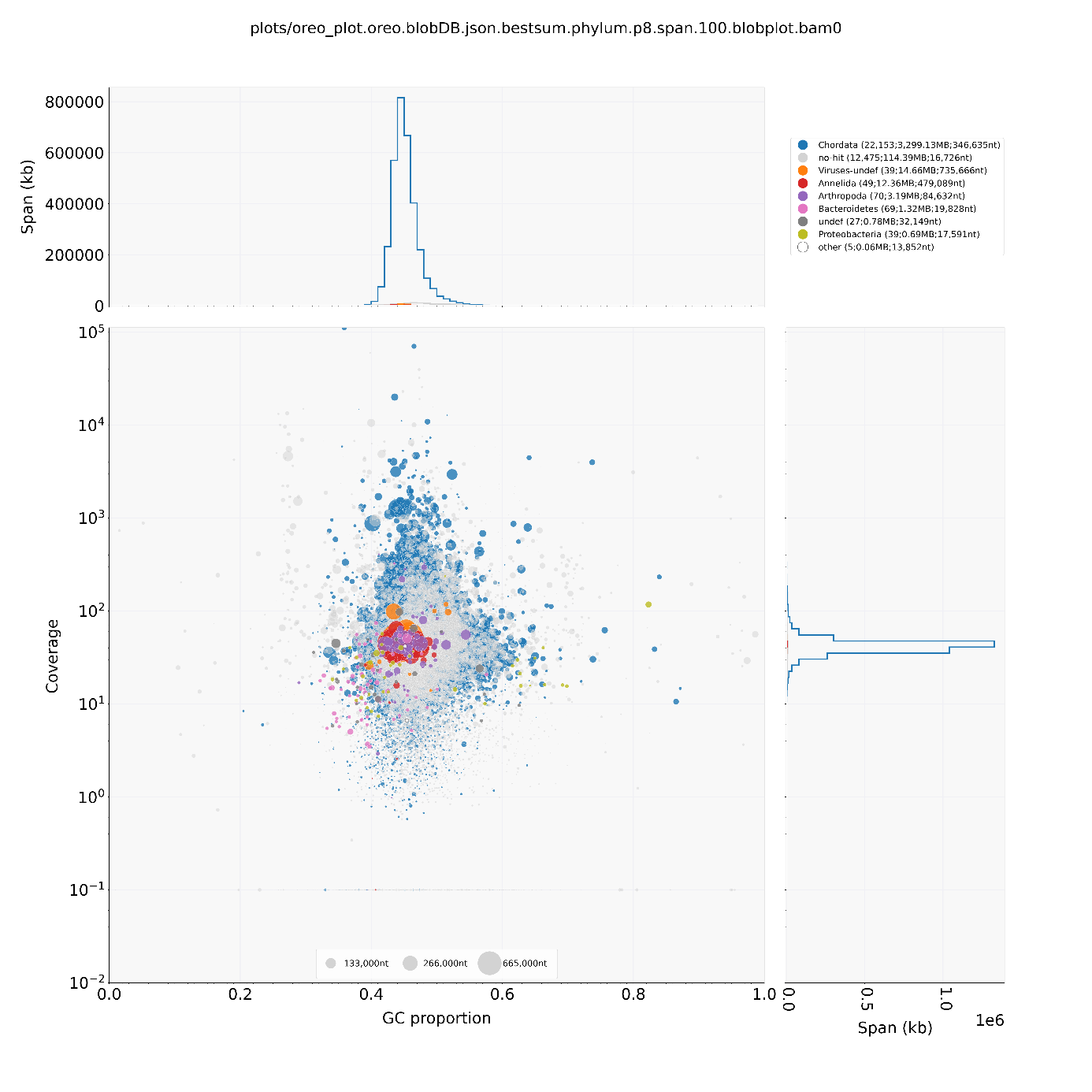


Figure S1: Blobtools plot showing little contamination in the reference assembly. All contigs form a single cluster and contigs assigned to taxa different from chordata likely represent false hits due to limited amphibian genomes available at the time of analysis.


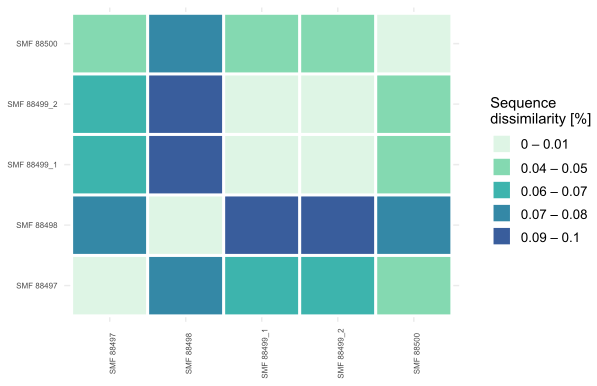


Figure S2: Raw sequence distances among mitochondrial genomes ignoring insertions and deletions. For individual SMF 88499 two different assemblies were reconstructed which differed exclusively by indels. As mitochondria differ between all specimens, all specimens had different mothers.
